# Effect of *Calotropis procera* (madar) and amprolium supplementation on parasitological parameters of broilers during mixed *Eimeria* species infection

**DOI:** 10.14202/vetworld.2017.864-868

**Published:** 2017-08-05

**Authors:** Sakshi Chauhan, V. S. Singh, Vipul Thakur

**Affiliations:** 1 Department of Veterinary Parasitology, College of Veterinary and Animal Sciences, G.B. Pant University of Agriculture and Technology, Pantnagar - 263 145, Uttarakhand, India; 2 Department of Veterinary Public Health and Epidemiology, Lala Lajpat Rai University of Veterinary and Animal Sciences, Hisar, Haryana, India

**Keywords:** amprolium, *Calotropis procera*, coccidiosis, parasitological parameters

## Abstract

**Aim:**

An experiment was conducted on day old 168 broiler chicks to study the effect of 0.4% as well as 0.2% *Calotropis procera* (madar) leaf powder and 0.0125% amprolium supplementation on parasitological parameters of broilers during mixed *Eimeria* species infection.

**Materials and Methods:**

Chicks were randomly divided into seven groups (I-VII) each with two replicates of 12 chicks. On 15^th^ day of experiment, broilers of Group II, IV, VI, and VII were infected with 50,000 sporulated oocysts of mixed *Eimeria* species. To evaluate the anticoccidial effect of different feed supplements percent fecal score, percent survival, percent weight gain, performance index (PI), average oocyst production, and percent reduction in oocyst production were calculated.

**Results:**

It was observed that amprolium supplementation had maximum anticoccidial effect as it gave the best efficacy in terms of all parameters, whereas supplementation of 0.4% madar leaf powder showed nonsignificant difference with amprolium for some parameters such as percent survival, percent weight gain, and PI.

**Conclusion:**

It can be concluded that madar (*C. procera*) leaf powder and amprolium had comparable activity against coccidiosis. Hence, madar leaf powder may be used for the prevention and control of mixed *Eimeria* spp. infection prevalent in field conditions.

## Introduction

Coccidiosis is one of the most dangerous diseases of poultry [1]. Coccidiosis is commonly called as red dysentery. It is caused by the intracellular protozoan parasite *Eimeria*, which undergoes its life cycle in the intestinal mucosa of the infected bird. Parasites present a monoxenous life cycle and are transmitted by the oral–fecal route. *Eimeria* may lead to a massive epithelial destruction. Consequently, the host may suffer with diarrhea, malabsorption, and poor weight gain [2].

Control of coccidiosis in modern intensive poultry production is based on the use of chemical coccidiostats in feed of poultry [1], but along with the problem of drug resistance, there are also food safety and public health concerns about drug residues in poultry products [3] which limit their use. Difficulties in tackling avian coccidiosis stimulated the scientists to explore further newer methods of control, and the natural products are being investigated to this effect [4]. Plant products (phytobiotics or phytogenics) could provide an alternative means of coccidia control to which resistance has not yet developed [5]. An important medicinal plant, *Calotropis procera*, has many pharmacological properties as antioxidant, antidiarrhea, anti-inflammatory, analgesic, antiulcer, antimicrobial, hepatoprotective, antipyretic, and antiparasitic activity [6]. Looking to the medicinal properties of madar present experiment was conducted to evaluate the anticoccidial effect of madar (*C. procera*) leaf powder in comparison with the standard anticoccidial Amprolium.

## Materials and Methods

### Ethical approval

This study was conducted after necessary approval from advisory and Institutional Animal Ethics Committee of College of Veterinary and Animal Sciences, G.B. Pant University of Agriculture and Technology, Pantnagar.

### Study area

Experiment was conducted in the College of Veterinary and Animal Sciences, G.B. Pant University of Agriculture and Technology, Pantnagar. For the experiment, 168, one-day-old commercial broiler chicks were randomly allocated to seven groups. The groups were designated as Groups I, II, III, IV, V, VI, and VII. Each group had two replicates having 12 chicks. The broilers were reared in electrically heated battery cages located in shed with 24 h light and ventilation. The cages were cleaned, washed and disinfected before each experiment and were fitted with separate feeders and waterers for different treatments. At the age of 5 days, vaccination against Ranikhet disease and at 12 days, vaccination against Gumboro disease was done using F_1_ strain and Georgia strain vaccine, respectively. Experiment was conducted for 30 days. Coccidiostat free feed for the broilers was brought from local poultry feed supplier. Feed given to all groups of broilers was isonitrogenic and isocaloric. Feed and drinking water were provided *ad-libitum* to the broilers during the entire experimental period. Broilers of Groups I and II were provided standard control diet without any supplement. In broilers of Groups III and IV, standard feed was supplemented with 0.0125% amprolium and broilers of Groups V and VI were provided with basal diet supplemented with 0.2% madar leaf powder. Broilers of Group VII were provided standard control diet supplemented with 0.4% madar leaf powder.

### Oocysts isolation

To isolate the oocysts of *Eimeria* sp., method described by Holdswort *et al*. [7] was used with few modifications. Oocysts were washed with water before use, and then the number of oocysts was counted in McMaster chamber [8]. *Eimeria* spp. mixed culture contained *Eimeria tenella* (80%), *Eimeria necatrix* (10%), *Eimeria acervulina* (6%), *E. maxima* (2%), and *Eimeria mitis* (2%), which were identified on the basis of guidelines of Levine [9]. At the age of 15 days, broilers of Groups II, IV, VI, and VII were infected by inoculating 1 ml suspension containing 50,000 sporulated oocysts of mixed *Eimeria* species directly in the pharynx, using a long nozzled 2 ml plastic pipette. Broilers of various groups, viz., I, III, and V were inoculated with 1 ml plain water.

### Evaluation of anticoccidial effect of different supplementations

To evaluate the anticoccidial effect of different feed supplements percent fecal score, percent survival, percent weight gain, performance index (PI), average oocyst production, and percent reduction in oocyst production were calculated in different groups.

Percent fecal score was calculated from 4^th^ to 10^th^ day postinfection as per the method described by Morehouse and Baron [10]. For this fecal collection trays of control as well as supplemented groups were divided into four parts and fecal score of each division was scored every day as 0 to +4. Percent fecal score for each supplemented infected group was determined as under:


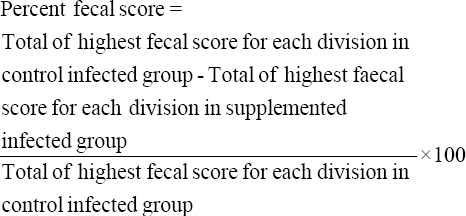


Percent fecal score thus obtained was rounded to nearest 5%.

To determine percent survival chicks were observed daily for the clinical manifestation and mortality after introduction of infection. Percent weight gain was calculated for each group from the formula given below:


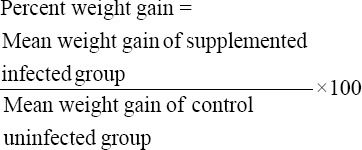


PI was calculated by adding percent survival, percent weight gain, and percent fecal score [10].

Fecal oocyst count was carried out from 4^th^ to 14^th^ day postinfection by collecting feces from each infected subgroup [8], and then average oocyst count was calculated. Percent reduction in oocyst production was calculated by the formula given below:


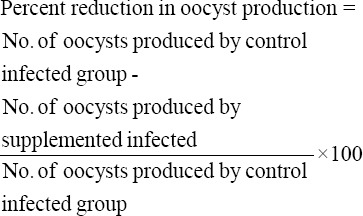


### Statistical analysis

All the observations recorded were subjected to statistical analysis using one-way ANOVA technique described by Snedecor and Cochran [11].

## Results

Parasitological parameters were absent in uninfected groups, so only infected groups were considered for statistical analysis.

**Figure-1:**
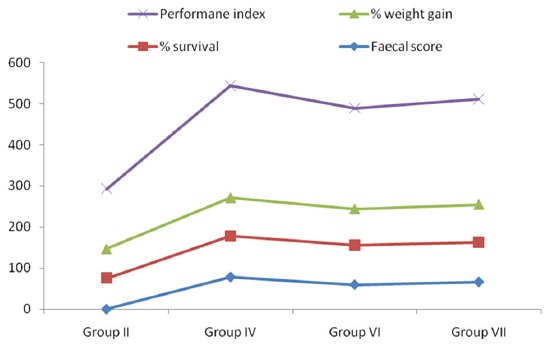
Effect of different supplementations on % faecal score, % survival, % weight gain and performance index in experimental broiler chicks.

Percent fecal score, percent survival, percent weight gain, and PI in all infected groups are presented in Table 1 and Figure-1.

**Table-1:** Effect of different supplementations on percent fecal score, percent survival, percent weight gain and performance index in experimental broiler chicks.

Groups		*Percent fecal score	*Percent survival	*Percent weight gain	*Performance index
II	IC	0.00±0.00^c^	75.00±00^b^	71.35±0.93^c^	146.35±0.93^c^
IV	IA	78.00±4.24^a^	100±0.00^a^	93.89±0.06^a^	271.89±4.18^a^
VI	IM-1	59.50±4.24^b^	95.84±5.89^a^	89.20±0.33^b^	244.54±10.46^b^
VII	IM-2	66.00±4.60^b^	95.84±5.89^a^	93.44±0.16^a^	255.03±10.33^ab^

* Significant;

a,b,c Means bearing different superscripts in a column differ significantly (p≤0.05). IC=Infected untreated control, IA=Infected supplemented with amprolium, IM-1=Infected supplemented with 0.2% madar leaf powder, IM-2=Infected supplemented with 0.4% madar leaf powder

Broilers of Group IV (78.00) had maximum and significantly higher percent fecal score, whereas Groups VI (59.50) and VII (66.00) had intermediate percent fecal score which differ nonsignificantly with each other.

Percent survival in broilers of infected untreated group, *i.e*., Group II (75.00) was lowest, whereas in Groups VI and VII percent survival was 95.84 and it was non-significantly lower than that of Group IV, which had cent percent survival. Similarly, Tipu *et al*. [12], Biu *et al*. [13], and Hady and Zaki [14] also observed lowest percent survival in control infected groups and increase in survival percent with the use of herbs. Biu *et al*. [13] observed that amprolium showed 100 percent survival rates against mixed *Eimeria* spp. oocysts.

Percent weight gain was highest in broilers of Group IV (93.89%) which differed nonsignificantly to broilers of Group VII (93.44%). Significantly lower and minimum percent weight gain was recorded in broilers of Group II (71.35%). Allen *et al*. [15] and Wang *et al*. [16] also reported increased body weight gain by dietary supplementation of herbal formulations in *Eimeria* spp. infected chickens. Abbas *et al*. [17] also found significantly higher (p≤0.05) weight gain in the groups infected with *E. tenella* and treated with 3% turmeric than that of infected control group.

PI of broilers of Group IV (271.89) was maximum, which was nonsignificantly higher than that of Group VII (255.03). PI of Group VII was also nonsignificantly different to Group VI (244.54), while significantly minimum PI was recorded in broilers of Group II (146.35). PI being the sum of percent fecal score, percent survival, and percent weight gain was improved due to the amprolium and herbal supplementation. Singh *et al*. [18] also recorded higher PI in sprouted mung supplemented group than control infected group.

**Figure-2:**
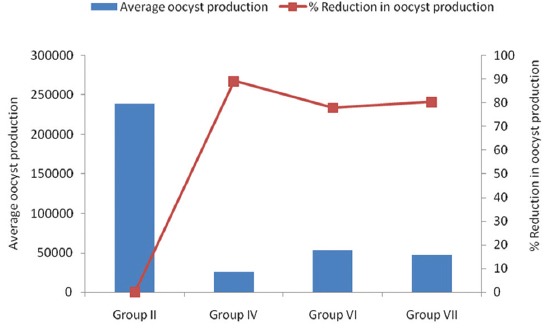
Effect of different supplementations on average oocyst production and % reduction in oocyst production in experimental broiler chicks.

Average oocyst production and percent reduction in percent oocyst production in all infected groups are presented in Table 2 and Figure-2.

**Table-2:** Effect of different supplementations on average oocyst production and % reduction in oocyst production in experimental broiler chicks.

Groups		*Average oocyst production	*Percent reduction in oocyst production
II	IC	239250±1343.50^a^	00±0.00^d^
IV	IA	25775±1025.31^d^	89.23±0.43^a^
VI	IM-1	52825±459.62^b^	77.92±0.19^c^
VII	IM-2	47150±212.13^c^	80.29±0.08^b^

* Significant;

a,b,c,d Means bearing different superscripts in a column differ significantly (p≤0.05). IC=Infected untreated control, IA=Infected supplemented with amprolium, IM-1=Infected supplemented with 0.2% madar leaf powder, IM-2=Infected supplemented with 0.4% madar leaf powder

Average oocyst production per gram of feces was 239250 in broilers of Group II, which was lower in all the supplemented groups and minimum for Group IV (25775). Groups VI (52825) and VII (47150) had intermediate average oocyst production, and these values were significant amid all the groups (p≤0.05). Oocyst production was significantly less in all supplemented groups than control infected group. Zaman *et al*. [19] also found that after *Eimeria* infection total oocyst production was lower in *C. procera* containing herbal complex treated and amprolium treated groups than control infected group. Mahmoud *et al*. [20] also observed the inhibiting effect of *C. procera* on oocyst production against *Eimeria ovinoidalis* in lambs.

There was no reduction in percent oocyst production in broilers of Group II, but in other groups, significant reduction in oocyst production was noticed. Group IV (89.23) had maximum percent reduction followed by Group VII (80.29) and Group VI (77.92). The present report is also in agreement with Tipu *et al*. [12] and Singh *et al*. [21] who used herbal anticoccidials and found similar results. Singh *et al*. [22] also noticed reduction in oocyst production with herbal vitamin E-selenium supplementation against mixed *Eimeria* spp. oocysts.

## Discussion

Plants produce a broad-spectrum variety of phytochemicals such as phenolics, polyacetylenes, alkaloids, polysaccharides, terpenoids, and essential oils with a large number of antimicrobial bioactivities [23]. Safe alternative to chemical anticoccidial drug is herbal products because they do not results to tissue residue and drug resistance [24]. The results of this study show the effectiveness of amprolium and madar for control of coccidiosis. Anticoccidial effect of madar leaf powder may be attributed to its anticoccidial property due to its saponin content, which act on the protozoan development by interacting with cholesterol present on the parasitic cell membrane and resulting into parasitic death [16,19] and phenols and flavonoids contents, which contribute to its antioxidant property and limit *Eimeria* induced damage to the intestinal wall during pro-inflammation reaction and resulting in less damage to the gut [25]. Antidiarrheal and antiulcer property of madar leaf powder also contributed to its anticoccidial effect [26,27]. El-Khtam *et al*. [28] also evaluated anticoccidial efficacy of *Allium sativum* powder and turmeric powder against coccidiosis in broilers which may be due to their antioxidant properties. *Carica papaya* suppresses coccidiosis by proteolytic destruction of *Eimeria* by papain and anti-inflammatory action by vitamin A [29]. Garlic and its sulfur compounds, i.e., allicin, alliin, ajoene, diallyl sulfide, dithiin, and allylcysteine, are reported to have broad antimicrobial activities. An *in vitro* study has shown that allicin inhibits sporulation of *E. tenella* effectively [30]. Green tea extracts significantly inhibited the sporulation process of coccidian oocysts. The selenium and polyphenolic compounds in green tea are thought to be active compounds to inactivate the enzymes responsible for coccidian sporulation [31].

## Conclusion

Considering the parasitological parameters, amprolium supplementation had maximum anticoccidial effect in terms of all parameters; however, supplementation of 0.4% madar leaf powder showed nonsignificant difference with amprolium for some parameters such as PI, percent survival, and percent weight gain. Hence, it can be concluded that anticoccidial effect showed by 0.4% madar leaf powder was comparable to that of amprolium and anticoccidial effect of 0.4% madar leaf powder was more than 0.2% madar leaf powder against mixed *Eimeria* spp. infection. Hence, madar leaf powder may be used for the prevention of mixed *Eimeria* spp. infection prevalent in field conditions.

## Authors’ Contributions

SC carried out the study. VSS helped in collection of samples, planning and supervision of the study. VT helped in data analysis and designing of the manuscript. All authors read and approved the manuscript.
